# Perivascular Adipose Tissue Attenuation on Computed Tomography beyond the Coronary Arteries. A Systematic Review

**DOI:** 10.3390/diagnostics11081495

**Published:** 2021-08-19

**Authors:** Domenico Tuttolomondo, Chiara Martini, Francesco Nicolini, Francesco Formica, Alessandro Pini, Francesco Secchi, Riccardo Volpi, Massimo De Filippo, Nicola Gaibazzi

**Affiliations:** 1 Department of Cardiology, Parma University Hospital, Via Gramsci 14, 43125 Parma, Italy; d.tuttolomondo@hotmail.it (D.T.); ngaibazzi@gmail.com (N.G.); 2 Department of Radiology, Parma University Hospital, Via Gramsci 14, 43125 Parma, Italy; 3 Department of Cardiac Surgery, Parma University Hospital, Via Gramsci 14, 43125 Parma, Italy; francesco.nicolini@unipr.it (F.N.); francesco.formica@unipr.it (F.F.); 4 Cardiovascular-Genetic Center, IRCCS Policlinico San Donato, 20097 Milano, Italy; alessandro.pini@grupposandonato.it; 5 Department of Radiology, IRCCS Policlinico San Donato, 20097 Milano, Italy; francesco.secchi@unimi.it; 6 Department of Biomedical Sciences for Health, Università degli Studi di Milano, 20133 Milano, Italy; 7 Department of Medicine and Surgery, Medical Clinic, University of Parma, Maggiore Hospital, Via Gramsci 14, 43125 Parma, Italy; riccardo.volpi@unipr.it; 8 Department of Medicine and Surgery, Section of Radiology, University of Parma, Maggiore Hospital, Via Gramsci 14, 43125 Parma, Italy; massimo.defilippo@unipr.it

**Keywords:** perivascular adipose tissue attenuation, computed tomography, stroke, transient ischemic attack, ascending aorta aneurysm, abdominal aortic aneurysms, vascular inflammation

## Abstract

(1) Background: Perivascular adipose tissue attenuation, measured with computed tomography imaging, is a marker of mean local vascular inflammation since it reflects the morphological changes of the fat tissue in direct contact with the vessel. This method is thoroughly validated in coronary arteries, but few studies have been performed in other vascular beds. The aim of the present study is to provide insight into the potential application of perivascular adipose tissue attenuation through computed tomography imaging in extra-coronary arteries. (2) Methods: A comprehensive search of the scientific literature published in the last 30 years (1990–2020) has been performed on Medline. (3) Results: A Medline databases search for titles, abstracts, and keywords returned 3251 records. After the exclusion of repetitions and the application of inclusion and exclusion criteria and abstract screening, 37 studies were selected for full-text evaluation. Three papers were finally included in the systematic review. Perivascular adipose tissue attenuation assessment was studied in the internal carotid artery, ascending thoracic aorta, and abdominal aorta. (4) Conclusions: Perivascular adipose tissue attenuation seems to be an applicable parameter in all investigated vascular beds, generally with good inter-observer reproducibility.

## 1. Introduction

Perivascular adipose tissue (PVAT) is a physiologic, specialized depot of fat that surrounds the blood vessels; it is currently interpreted as a paracrine organ that produces a wide range of biologically active molecules, which may have a profound influence on the vasculature itself [1,2]. Perivascular adipose tissue attenuation, measured with computed tomography (CT) imaging, is a non-morphological analysis that was first applied to coronary arteries. It is a marker of mean local vascular inflammation since it reflects the histopathological alterations of PVAT in the volume of the fat tissue in contact with the vessel. Pericoronary adipose tissue (PCAT) attenuation is useful for the detection of coronary inflammation, either using a binary cut-off (less than −70 Hounsfield units is typically considered inflammation in the coronary artery bed) or being capable to detect dynamic changes—for example, in the setting of vulnerable and inflamed atherosclerotic plaques during or after acute coronary syndromes. It has been demonstrated that coronary vessels exert paracrine effects on the surrounding PCAT, thus affecting local intracellular lipid accumulation and determining the morphological changes of adipocytes, both in terms of their size and lipid content, and also the reverse from the PCAT to the vessel. Such morphological changes can be detected through the measurement of PCAT attenuation; it may be also useful to improve cardiac risk prediction through coronary CT since it provides a quantitative measure of coronary inflammation. The role of inflammation in the pathogenesis and progression of ischemic cardiovascular disease is now well known, to the point that the use of immunomodulatory/anti-inflammatory drugs, such as Canakinumab and Colchicine, in patients with ischemic heart disease reduces the risk of recurrent cardiovascular events [3,4,5]. Higher PCAT attenuation values can predict increased cardiac mortality [6]. 

The aim of the present systematic review is to provide insight into the potential application of PVAT attenuation through CT imaging in *extra-coronary* vascular beds, which have rarely been addressed in clinical studies. 

Therefore, in the current review, we ask the following question: “Is PVAT attenuation by CT imaging potentially useful in vascular diseases other than the coronary bed?” This question has been formulated according to the Patient, Intervention, Comparison, Outcome (PICO) worksheet.

## 2. Materials and Methods

**Figure 1:**
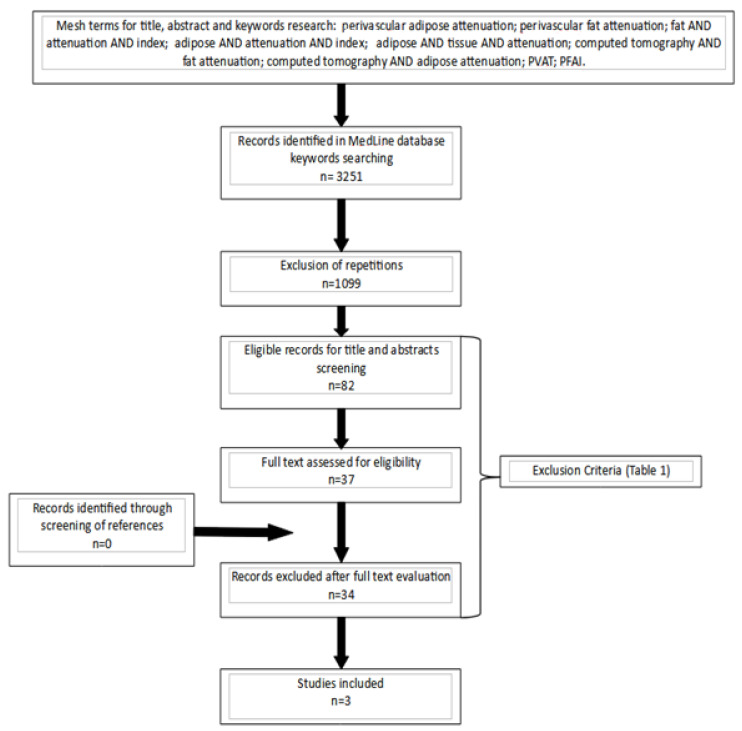
Flow-chart diagram for the selection of the 3 papers included in the review.

The present systematic review has been designed following the guidelines of the Preferred Reporting Item for Systematic Reviews and Meta-analysis (PRISMA) checklist. A comprehensive search of the scientific English literature published in the last 30 years (1990–2020) has been performed up to November 2020. The following searches have been performed on Medline: “perivascular adipose attenuation”; “perivascular fat attenuation”; “fat” AND “attenuation” AND “index”; “adipose” AND “attenuation” AND “index”; “adipose” AND “tissue” AND “attenuation”; “computed tomography” AND “fat attenuation”; “computed tomography” AND “adipose attenuation”; “PVAT”; “PFAI”. After a software-aided elimination of duplicates (using EndNote X9^©^ software, Clarivate Analytics, Clarivate, Philadelphia, PA 19130, USA), records were evaluated through a first-level screening (i.e., title evaluation). At this level, case reports, conference proceedings, personal communications, letters to the editor, and reviews were excluded. Papers selected for second-level screening underwent a careful analysis of the abstract and—in case of potential interest—were considered eligible for full-text evaluation. All references of review articles resulting from the research were screened, in order to identify potentially missed studies. This approach was also applied to the reference sections of papers that were determined to be eligible for selection. The search flow is summarized in Figure 1.

Final eligibility was assessed according to the following exclusion and inclusion criteria: for inclusion, studies that were performed on humans with PVAT attenuation and were assessed through CT imaging; for exclusion: studies not written in the English language. A comprehensive recap of the selection criteria is provided in Table 1.

### Data Extraction, Quality Assessment, and Critical Appraisal

Data extracted from each study were summarized into an Excel^©^ table (Office Suite, Microsoft Corp, Redmond, Washington, USA). Such data included: the title, authors, year of publication, the number of involved patients, the vascular site of the PVAT attenuation measurement through CT imaging, the endpoint of the study, and the statistical significance threshold. Two independent reviewers separately evaluated all papers using the National Institutes of Health’s (NIH’s) National Heart, Lung and Blood Institute Study Quality Assessment Tool (National Heart Lung & Blood Institutes, Bethesda, MD 20892, USA). Specifically, the questionnaire for case–control studies within this tool was utilized (https://www.nhlbi.nih.gov/health-topics/study-quality-assessment-tools, accessed on 10 August 2021). Such tools were specifically developed for each type of study (controlled intervention studies, systematic reviews with meta-analyses, observational cohort and cross-sectional studies, case–control studies, before–after studies without a control group, and case series studies). The questionnaire is designed to help the investigator identify potential flaws caused by study design and conduction. Questions 9, 10, and 11, which pertain to exposure/risk, are not applicable in case–control studies.

The critical appraisal of studies has been performed through the assignment of a score ranging from 0 to 100%, based on the percentage of “yes” answers on the overall number of answers given. Studies having an 80–100% score were labeled as having “good” quality, those ranging from 50 to 70% were “fair”, and studies scoring less than 50% were defined as “poor” quality studies. No discrepancies in score assignment have emerged.

The level of evidence was assessed using the Oxford Center for Evidence-Based Medicine’s classification levels for diagnosis (2011).

## 3. Results

The research algorithm is summarized in Figure 1. The search of the Medline databases for titles, abstracts, and keywords returned 3251 records. After the exclusion of repetitions and the application of inclusion and exclusion criteria, 82 records were considered eligible for title and abstract screening. Full-text evaluations were performed for 37 records, and 34 records were discarded at this level. No records were included from article references cross-checking. Eventually, three papers were finally included in the review [7,8,9]. 

### 3.1. Quality Assessment

**Figure 2:**
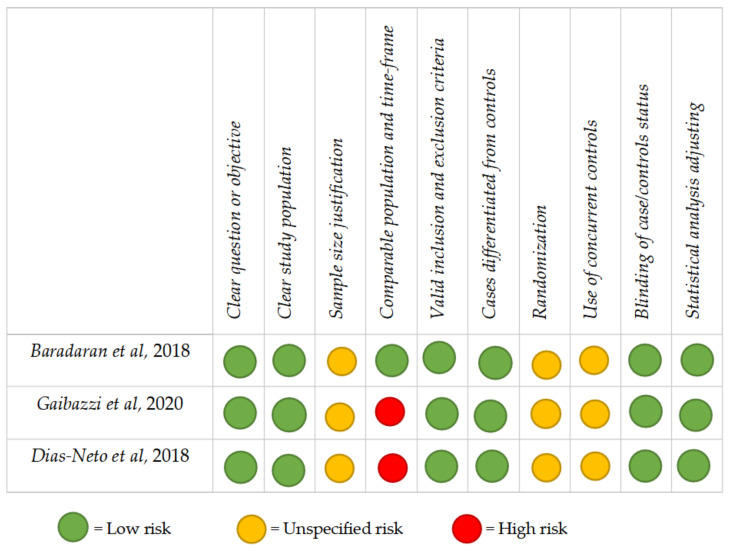
Critical appraisal, including the main potential risk of bias and quality score.

No disagreement between the two reviewers occurred. No “good” or “poor” quality papers were identified in the present systematic review. All studies were classified as having “intermediate” quality. Potentially fatal flaws have emerged from the assessment process: studies 2 and 3 (66%) did not describe a comparable population for recruitment or the correspondence of the timeframe of recruitment. In general, the most frequently encountered potential risks of bias (ROBs) were the absence of concurrent controls (3 studies; 100%); sample size justification (3 studies; 100%); and randomization (3 studies; 100%). The critical appraisal of the selected papers is summarized in Figure 2.

### 3.2. Level of Evidence

The application of the Oxford Center for Evidence-based Medicine (CEMB) guidelines highlighted that all the selected papers have a low level of evidence (4 on a scale of 5, with the fifth level being the lowest) because of their case–control design. 

### 3.3. Perivascular Adipose Tissue Attenuation

Overall, the evaluation of PVAT attenuation was performed in three vascular beds, while inter-observer reproducibility has been executed in only two of these (study 1 and study 3). A comprehensive recap the contents of the selected studies is provided in Table 2.

### 3.4. Carotid Artery

Study 1 is a single-center, case–control study and includes patients with monolateral internal carotid artery (ICA) stenosis ≥50% up to 99%. Patients were divided into 2 groups: a symptomatic group, with 42 patients affected by a cerebral ischemic event (29 patients had a stroke and 13 had a transient ischemic attack (TIA)) and an asymptomatic group (*n* = 52) with no cerebral ischemic events [7]. The pericarotid fat density of the stenotic ICA in symptomatic patients was significantly higher compared with the similarly diseased ICA in asymptomatic patients (*p* = 0.001). When comparing the fat density around the nonstenotic ICA, there was no significant difference between the mean Hounsfield units (HU) measured in symptomatic patients compared with asymptomatic patients (*p* = 0.198). A within-subjects comparison analysis was also performed, with the pericarotid mean fat density being significantly increased around the stenotic ICA versus the nonstenotic ICA (*p* < 0.001); this difference was maintained in the subgroup analysis, in both symptomatic patients (*p* < 0.001) and asymptomatic patients (*p* = 0.001). An inter-rater reliability analysis for the measurement of fat density was performed in a subset of 40 ICAs, showing good interclass coefficients for mean and maximum pericarotid fat density, of 0.83 (95% CI 0.63 to 0.93) and 0.94 (95% CI 0.87 to 0.98), respectively.

### 3.5. Ascending Thoracic Aorta

Study 2 is a single-center, retrospective case–control study and includes two groups: patients with ascending aorta aneurysm (AAA) (*n* = 160) and individuals without apparent aortic disease (*n* = 80) [8]. The mean PVAT attenuation value (HU) was higher in patients with AAA compared with the control group (*p* < 0.0001). A significant correlation between ascending aorta diameter and PVAT attenuation was also observed, but only in the AAA group (*p* < 0.0001). The inter-observer reproducibility of PVAT attenuation measurement in this study was not reported.

### 3.6. Abdominal Aorta

Study 3 is a multicenter, retrospective case–control study and includes three groups of patients: those with nontreated asymptomatic abdominal aortic aneurysms (AbAA) (*n* = 140), aorto-iliac occlusive disease (AIOD) (*n* = 104), and individuals without evidence of aortic pathology (*n* = 97) [9]. The average abdominal aortic PVAT density was compared between AbAA patients, AIOD patients, and a control group. Additionally, the PVAT densities in two aortic regions of the same patient were compared to each other; intra-individual PVAT assessment was performed to explore regional differences in PVAT density within the same individuals by comparing diseased and non-diseased aortic segments. The patients with AIOD showed lower PVAT density than other groups (*p* < 0.001). Nevertheless, this finding was not statistically significant when a linear regression analysis model was built. In the control group, the intra-individual PVAT attenuation comparison was not statistically significant (*p* = 0.123), meaning that PVAT density showed no difference between the two regions tested. In patients with AbAA, the intra-individual PVAT density was instead significantly different (*p* = 0.001). As expected from these findings, the comparison of the intra-individual PVAT attenuation difference within each patient among the three groups showed a significantly higher intra-individual PVAT attenuation difference in patients with AbAA than the other two groups, and the association persisted after adjustments for cardiovascular risk factors and diseases and the measurement of other fat compartments (*p* = 0.006). The inter-observer reproducibility of adipose tissue measurements was assessed by two independent readers in 50 randomly selected patients. The intraclass correlation coefficient and the respective 95% CI of PVAT densities is 0.97 (0.95–0.99).

## 4. Discussion

The role of inflammation appears to be central in the etiopathogenesis of vascular disease in multiple beds. The serum C-reactive protein is a marker of systemic inflammation and has been associated with an increased risk of stroke and carotid atherosclerotic plaque instability [10,11]. Interleukin-1β (IL-1β) is upregulated in AAA and functions by establishing a cycle of inflammation through the binding of its receptor, releasing multiple proinflammatory cytokines, including more IL-1β in positive feedback [12,13]. Inflammation also plays a key role in abdominal aortic aneurysms, where myeloperoxidase, tissue-type plasminogen activators, and cystatin-B levels were significantly associated with AbAA growth, even after adjusting for baseline AbAA diameter [14].

The clinical use of systemic markers of inflammation has the limitation that they are unable to pinpoint specific vascular beds, while PVAT attenuation allows for overcoming this limitation by assessing the degree of local vascular inflammation. The relationship between the vascular wall and the surrounding adipose tissue is complex and likely bidirectional [15,16]. The role of localized inflammation in coronary atherosclerotic disease is now well known. The predictivity of the degree of coronary perivascular attenuation has already been demonstrated in terms of cardiovascular mortality and all-cause mortality—and even incrementally to known prognostic radiological markers, such as: I) “spotty calcification”, II) “low-attenuation plaque”, III) “positive remodeling” and IV) “napkin-ring sign” [17]. The role of PVAT density as a local inflammatory marker, and the possibility of measuring changes noninvasively on repeated CT exams, should be contextualized in this setting. 

The aim of this systematic review is to assess the potential use of these vascular measurements beyond the coronary arteries. Study 1, overall, suggests that there is increased inflammation in the fat surrounding symptomatic ICAs in patients with ischemic events secondary to carotid disease and that this local inflammation is likely independent of any generalized systemic inflammation, given the relatively lower density of pericarotid fat in the nonstenotic ICA [7]. Similar to what happens in coronary arteries, these findings suggest that the presence of significant carotid artery stenosis is associated with increased perivascular fat inflammation, regardless of the presence of ipsilateral neurologic symptoms [17,18]. Study 2 reports that AAAs are associated with higher-density PVAT around the ascending aorta, and such higher density appears to be secondary to an elevated perivascular inflammatory state [8]. In study 3, the presence of an AbAA was an independent predictor of increased intra-individual PVAT attenuation differences. Furthermore, intra-individual PVAT differences were positively associated with aortic volume [9]. These findings suggest that PVAT plays a role in AbAA pathophysiology via local mechanisms. 

We think that the potential usefulness of a local inflammatory marker to assess multiple vascular beds, specifically measurable with simple CT techniques, is definitely worth the design and performance of new ad-hoc studies following the best methodological scientific rules. In particular, the lack of a prospective design and appropriate control group was a common key limitation in the only three studies reporting on non-coronary vascular beds that were identified and included in the current metanalysis.

## 5. Conclusions

Perivascular adipose tissue attenuation through computed tomography imaging has emerged as an applicable parameter in the carotid artery, in the ascending and abdominal aorta, generally with good inter-observer reproducibility. At the moment, with the typical limitations of retrospective studies, the existing studies do encourage further investigations regarding a prognostic role of such fat attenuation measurement in beds other than the coronary arteries, and they suggest the need for a more rigorous, blinded, and prospective study design for future studies.

## Figures and Tables

**Table 1:** Inclusion and exclusion criteria.

Inclusion Criteria	Exclusion Criteria
Studies published up to November 2020	Studies different from the case–control setting
Studies performed on humans with the execution of perivascular adipose tissue attenuation through computed tomography imaging	Publication type: case reports, conference proceedings, personal communications, letters to the editor, and reviews
	Studies not written in the English language

**Table 2:** General characteristics of the selected papers, the results, and their statistical significance.

Authors	Aim	Subjects	Exclusion Criteria	Statistical Significance
Baradaran et al. [8]	To evaluate the association between pericarotid inflammation, measured through the density of carotid PVAT on CT, with stroke and TIA.	94 patients with unilateral ICA stenosis ≥50% to 99%: 42 with stroke or TIA; 52 asymptomatic patients.	Simultaneous bilateral anterior circulation stroke or TIA, bilateral extracranial ICA stenosis.	In the between-subject analysis of stenotic ICAs, symptomatic patients had higher mean pericarotid fat density compared with asymptomatic patients (*p* = 0.009) without significant difference in non-stenotic ICAs (*p* = 0.198). Within-subject comparison showed increased density in stenotic ICA versus non-stenotic ICA (*p* < 0.0001).
Gaibazzi et al. [9]	To evaluate differences in periaortic inflammation, measured through ascending aorta PVAT attenuation on CT, among patients with AAA and controls.	240 subjects: 160 AAA; 80 HC.	Myocardial infarction, heart valvular or ascending aorta surgery, coronary Revascularization, or another type of percutaneous intervention, inflammatory disease, infective disease, or active neoplasia.	In the overall study population, simple linear regression showed a significant positive correlation between the PVAT attenuation and the maximum diameter of the ascending aorta (*p* < 0.0001).
Dias-Neto et al. [10]	To evaluate differences in periaortic inflammation, measured through abdominal aortic PVAT attenuation on CT, among patients with AbAA, AIOD, and patients without aortic disease.	341 subjects: 140 AbAA; 104 AIOD; 97 HC.	Ruptured AbAA, symptomatic AbAA, inflammatory AbAA, clinical symptoms suggesting aortic inflammation or inflammatory aspects on CT, previous abdominal aortic intervention, active neoplasia, active infection, or incomplete CT scans.	AbAA patients presented higher intra-individual PVAT differences with higher PVAT density around the aneurysm sac than the healthy neck (*p* < 0.006).

PVAT, perivascular adipose tissue; CT, computed tomography; TIA, transient ischemic attack; ICA, internal carotid artery; AAA, ascending aorta aneurysm; HC, healthy controls; AbAA, abdominal aortic aneurysms; AIOD, aorto-iliac occlusive disease.

## Data Availibility Statement

https://www.nlm.nih.gov/medline/medline_overview.html (accessed on 10 August 2021).
